# Sudden Cardiac Death in Pregnant Women—Literature Review and Autopsy Findings

**DOI:** 10.3390/diagnostics15091108

**Published:** 2025-04-27

**Authors:** Ioana Radu, Anca Otilia Farcas, Laura Cimpan, Corina-Lacramioara Platon, Victoria Nyulas, Bogdan Andrei Suciu, Ioana Hălmaciu, Carmen Corina Radu, Klara Brînzaniuc

**Affiliations:** 1 Doctoral School of Medicine and Pharmacy, George Emil Palade University of Medicine, Pharmacy, Science, and Technology of Targu Mures, 540142 Targu Mures, Romania; ioanaradu888@gmail.com; 2 Department of Forensic Medicine, Emergency County Hospital, “Constantin Opris” Baia Mare, 430031 Baia Mare, Romania; laura10cimpan@gmail.com (L.C.); corinaplaton.2024@gmail.com (C.-L.P.); 3 Department of Cell Biology, George Emil Palade University of Medicine, Pharmacy, Science, and Technology of Targu Mures, 540139 Targu Mures, Romania; 4 Department of Informatics and Medical Biostatistics, University of Medicine Pharmacy Science and Technology George Emil Palade of Târgu Mureș, 540142 Targu Mures, Romania; victoria.rus@umfst.ro; 5 Department of Anatomy, George Emil Palade University of Medicine, Pharmacy, Science, and Technology of Targu Mures, 540139 Targu Mures, Romania; bogdan.suciu@umfst.ro (B.A.S.); klara.brinzaniuc@umfst.ro (K.B.); 6 Department of Radiology, George Emil Palade University of Medicine, Pharmacy, Science, and Technology of Targu Mures, 540139 Targu Mures, Romania; ioana.halmaciu@umfst.ro; 7 Department of Radiology, Mures County Emergency Hospital, 540136 Targu Mures, Romania; 8 Department of Forensic Medicine, George Emil Palade University of Medicine, Pharmacy, Science, and Technology of Targu Mures, 540139 Targu Mures, Romania; carmen.radu@umfst.ro; 9 Institute of Forensic Medicine, 540141 Targu Mures, Romania

**Keywords:** sudden maternal death, pregnancy, autopsy, molecular biology, fibroelastosis, sudden cardiac death, borderline myocarditis

## Abstract

Cardiovascular diseases increase among pregnant women and complicate 1–4% of pregnancies worldwide. The incidence of maternal deaths due to cardiovascular causes has increased dramatically, rising from 3% three decades ago to 15% in recent years. The aim of this study is to provide a comprehensive overview of the current status of knowledge in sudden maternal death (SMD) described in the literature and to present two cases of autopsy findings in sudden cardiac death in pregnant women. Among the most common causes of sudden maternal deaths are peripartum cardiomyopathies, aortic dissection, acute myocardial infarction, arrhythmias, ischemic heart disease, and coronary artery dissection, and among the less common causes, we list coronary artery dissection, congenital heart diseases, valvulopathies, hypertension, fibroelastosis, and borderline myocarditis. The Centers for Disease Control and Prevention (CDC) reported that over 80% of pregnancy-related deaths were preventable. To reduce the number of maternal deaths caused by cardiovascular diseases, the implementation of specialized multidisciplinary teams has been proposed. Molecular biology techniques are proving their effectiveness in forensic medicine. PCR or DNA sequencing can be utilized in “molecular autopsy”, which holds particular value in cases of sudden death where the forensic autopsy is negative but there is a suspicion that death was caused by arrhythmia. Susceptibility genes can be analyzed, such as KCNQ1, KCNH2, KCNE1, and KCNE2, which are involved in long QT syndrome, the RYR2 gene implicated in catecholaminergic polymorphic ventricular tachycardia type 1, or the SCN5A gene associated with Brugada syndrome. Early identification of risk factors involved in sudden maternal death prenatally and during pregnancy is essential. At the same time, genetic determinations and molecular biology techniques are absolutely necessary to prevent the occurrence of sudden deaths among close relatives.

## 1. Introduction

Cardiovascular diseases have been experiencing a significant increase both in the general population and among pregnant women and are estimated to complicate 1–4% of pregnancies worldwide. These conditions represent a major contributing factor to maternal mortality in both developed and developing countries [1,2,3].

Sudden cardiac death (SCD) is a major public health concern that affects 4–5 million cases annually according to some authors and 6–9 million cases according to others, representing 10–15% of all deaths worldwide. However, its exact incidence in young women during pregnancy or immediately postpartum is unknown [4,5,6,7,8].

In developed countries, the incidence of maternal deaths due to cardiovascular causes has increased dramatically, rising from 3% three decades ago to 15% in recent years. A plausible explanation for this alarming trend is the increase in maternal age [9,10]. In Romania, the maternal mortality rate remains high (10 per 100,000 live births in 2020). However, this rate includes all causes of maternal death, not solely those attributable to sudden cardiac death [11].

Sudden deaths during pregnancy are of particular interest, so it is extremely important to know their potential causes, as well as the circumstances that caused them. Among the most common causes of sudden deaths in the prenatal and postnatal period are cardiovascular conditions (sudden arrhythmic death syndrome, dissection of the coronary arteries or great vessels, and valvular or congenital heart diseases), peripartum cardiomyopathies, acute myocardial infarction, ischemic heart disease, and cerebrovascular accidents, as well as complications that can frequently occur in pregnancy (thromboembolism, amniotic fluid embolism, and infections) [2,9,12].

Sudden maternal death is defined as death due to an unknown or previously uninvestigated cardiovascular condition [13,14]. Death occurs suddenly and unexpectedly in women who do not have a known cardiovascular disease, are in apparent health, and are asymptomatic, or at most one hour after the onset of premonitory symptoms in the presence of witnesses [15,16,17]. SCD can occur in any age group and sex, but in young people and even more so in pregnant women or young mothers, it is perceived as an extremely tragic event.

Although cardiomyopathies, hypertension, and undiagnosed pulmonary hypertension represent major health concerns, standardized investigations for identifying potential cardiac diseases are not routinely conducted in pregnant women. During pregnancy, attention is mainly focused on the fetus, and investigations, even in pregnant women over 35 years of age, mainly aim to identify genetic disorders that may affect the fetus.

The aim of this study is to provide a comprehensive overview of the current status of knowledge in sudden maternal death (SMD) described in the literature and to present two cases of autopsy findings in sudden cardiac death of pregnant women registered in the Maramureș Forensic Medicine Service, with extremely rare causes of death found in the literature.

We studied the literature by accessing PubMed and Web of Science to find the most recent research on sudden maternal deaths. The most relevant articles published in the last 10 years were selected, using results from searching for keywords such as “sudden maternal death”, “sudden cardiac death”, “pregnant women”, and “autopsy findings”.

We also studied the forensic autopsy reports from the Maramureș County Forensic Medicine Service from 2019 to 2024 to identify sudden deaths in pregnant women, with two cases of sudden death in pregnant women being recorded, both occurring in the last trimester of pregnancy.

## 2. Relevant Sections

### 2.1. Causes of SCD in Pregnant Women

Literature data on sudden maternal deaths are extremely poor globally, and in Romania, there are no current large studies on this topic. A study conducted in France shows that one in six maternal deaths is sudden [18]. According to the largest study conducted on a group of 80 maternal deaths by Krexi D. and Sheppard M.N. [19] in the UK, the most common cause of maternal death was SADS (sudden arrhythmic death syndrome) (53.75%), followed by cardiomyopathies (13.8%).

Cardiomyopathies are defined as an acquired alteration of myocardial structure and function in the absence of congenital heart disease or coronary or valvular pathology [20,21]. They are classified into the following types: those with a predominantly genetic predisposition, hypertrophic cardiomyopathy (HCM) and arrhythmogenic right ventricular cardiomyopathy (ARVC), and those with both genetic and acquired transmission, restrictive cardiomyopathy (RCM) and dilated cardiomyopathy (DCM). The most common form during pregnancy is peripartum cardiomyopathy (PPCM), which occurs in the last month of pregnancy or in the first 5 months postpartum, being rather a diagnosis of exclusion in women with heart failure who present with systolic dysfunction (LVEF < 45%). This leads to a highly variable maternal mortality from <2% to 50% [22,23,24,25].

The etiology of PPCM is multifactorial and is not fully understood. In addition to the hemodynamic stress of pregnancy, autoimmune processes, nutritional deficiencies, and infectious agents are believed to be contributing factors in the development of PPCM [26,27,28]. Obesity, race, maternal age (over 30 years), multiparity, and chronic arterial hypertension are also considered risk factors [29,30,31,32].

The symptoms of PPCM are nonspecific, found in any type of heart failure, and some are also present even in a normal pregnancy. Thus, the predominant symptom is dyspnea, followed by orthopnea, cough, palpitations, chest pain, paroxysmal nocturnal dyspnea, and hemoptysis; as such, an immediate and correct diagnosis is difficult to establish, and the treatment remains a challenge due to the fetal risk of the medication [33,34,35].

Arrhythmogenic right ventricular cardiomyopathy is a cardiac condition that affects the young or middle-aged population, and consequently is also encountered in pregnant women, characterized by the thinning and replacement of fibro-adipose tissue in the right ventricular wall with the subsequent dilation of the cavity [36,37]. In some cases of ARVC, nonspecific symptoms such as palpitations accompanied by chest discomfort may appear, but there are also situations in which these symptoms are absent, leading to SCD [38].

Aortic dissection is another potential cause of maternal death due to hormonal changes during pregnancy, with increased heart rate and volume, being responsible for 3–14% of maternal cardiac deaths [39,40]. At the same time, hormonal changes caused by estrogen and progesterone can produce changes in the structure of the aortic wall through a reduction in acid mucopolysaccharides and, implicitly, fragmentation of reticulin due to disorganization of elastic fibers, causing aneurysmal dilatation followed in some cases by dissection [41,42]. Symptoms that precede aortic dissection are anterior or posterior chest pain, sometimes radiating to the arm, shortness of breath, and circulatory collapse.

Acute myocardial infarction has a low incidence in pregnancy compared to other cardiovascular diseases, reported as being between 3 and 100 per 100,000 live births, but the incidence is extremely variable, estimated, according to the study by Bush et al. [43] conducted in the UK, at 0.7 per 100,000 mothers (95% CI 0.5–1.1) and, in the James et al. study [44] conducted in the USA, at 6.6 per 100,000 women, but it requires considerable attention due to its alarming lethal potential, with the risk of perinatal morbidity and mortality increasing worldwide [45,46,47]. In the absence of cardiovascular diseases pre-existing pregnancy and risk factors specific to the general population (smoking, obesity, metabolic syndrome, sedentary lifestyle, diabetes mellitus, and genetic predispositions), AMI, according to studies, has a 3–4 times higher incidence in pregnancy due to coagulative changes (hypercoagulability), hypervolemia, thrombophilia, eclampsia, preeclampsia, blood transfusions, and multiparity being favorable factors [48,49,50,51,52,53]. Maternal mortality associated with AMI during pregnancy is reported at 37% [44,54].

Unlike the general female population, in which the most common cause of AMI is stenosing coronary atherosclerosis with or without intracoronary thrombus, in pregnancy-associated AMI, the non-atherosclerotic etiology predominates, i.e., spontaneous coronary artery dissection, with an incidence of 27–43% of deaths caused by AMI in pregnant women. Another non-atherosclerotic etiology is intracoronary thrombus without atherosclerosis, with a prevalence of 8–17%, followed by coronary artery spasm, with an incidence of 2% [55,56]. Acute myocardial infarction can occur throughout pregnancy and postpartum, but most frequently occurs in the third trimester of pregnancy, the diagnosis being based on symptoms, electrocardiographic changes, and cardiac serum markers [57].

One of the most common etiologies of acute myocardial infarction during pregnancy is represented by spontaneous coronary artery dissection (SCAD) [58,59,60]. This predominantly affects pregnant women in the third trimester of pregnancy and in the first month postpartum [61]. There are no exact data on the incidence of SCAD in pregnant women, but, according to the study by Faden et al. [62], it has a prevalence of 1.8 per 100,000 pregnancies [63]. This is defined as a separation of non-traumatic, non-iatrogenic, and non-atherosclerotic etiology of the walls of the coronary arteries with the creation of a false lumen and the formation of an intramural hematoma that compresses the arterial lumen [64,65,66]. SCAD is responsible for about 40% of myocardial infarctions occurring during pregnancy, causing an uncharacteristic symptomatology with chest pain, followed in a short time by cardio-respiratory arrest and death [67].

Spontaneous coronary and aortic dissections are serious events that can occur due to genetic conditions that affect the structure and strength of blood vessels. Several genetic syndromes are associated with an increased risk of aortic and other great vessel dissection, including Marfan syndrome, Loeys–Dietz syndrome, Ehlers–Danlos syndrome, Alport syndrome, and other genetic conditions such as fibromuscular dysplasia.

### 2.2. Etiology and Risk Factors of Sudden Maternal Death

Data regarding sudden death in pregnant women are scarce in the literature, and large-scale studies addressing this issue are limited. Sophia Braund et al. [18], in an extensive study, analyzed the causes of maternal deaths recorded in France over a six-year period. The results of the study revealed a total of 10.3 maternal deaths per 100,000 live births, of which 1.7 deaths per 100,000 live births were classified as sudden maternal deaths. Regarding the identification of the cause of maternal death, in 38.6% of cases, the cause remained undetermined, while 12% of deaths were attributed to cardiovascular diseases. Other etiologies of sudden maternal death recorded in the study included pulmonary thromboembolism in 23% of cases and amniotic fluid embolism in 17% of sudden deaths. Other causes of death encountered were epilepsy in 6% of cases, stroke, or air embolism.

In the USA, the second leading cause of peripartum mortality is amniotic fluid embolism, which is also the leading cause of peripartum cardiac arrest, with a reported incidence between 1.9 and 6.1 per 100,000 births [68]. Amniotic fluid embolism leads to fulminant deterioration of cardio-circulatory function accompanied by disseminated intravascular coagulopathy, caused by an anaphylactoid reaction due to the presence of amniotic fluid and fetal tissues (hair, meconium, squamous epithelium, etc.) in the maternal pulmonary circulation [69]. Risk factors for amniotic fluid embolism include advanced maternal age, multiparity, male fetuses, polyhydramnios, cerebrovascular disease, and cardiovascular disease [70].

In their study, Krexi D. et al. [19] investigated the cardiovascular causes that led to sudden maternal deaths recorded in the UK. Of the 80 cases of maternal sudden cardiac death, 50% were recorded during pregnancy and 50% postpartum. In the study, the mean age was 30 ± 7 years, with the minimum age being 16 years and the maximum age being 43 years. The authors reported 30% of maternal deaths in women over 35 years of age, whereas 42.86% (21 cases) of the cases for which BMI was available had a BMI ≥ 30, and 16.33% (8 cases) had a BMI between 25 and 30 (overweight). In 53.75% of cases (43 deaths), the heart did not present morphological changes. In 13.80% (11 deaths), cardiomyopathies were detected, of which seven were dilated cardiomyopathies. Other cardiac causes of death recorded in the study were aortic dissection, coronary dissection, congenital heart diseases, valvular diseases, hypertension, left ventricular hypertrophy, myocardial fibrosis, and Libman–Sacks endocarditis.

In the USA, as well as in other developed countries (high-income countries), cardiovascular diseases are the main cause of mortality among pregnancy-related deaths, with 25% of maternal deaths recorded in California caused by cardiovascular diseases. The most common etiology reported by Hameed AB et al. [71] was cardiomyopathy, causing 65.62% of the sudden cardiac deaths recorded in their study, with dilated cardiomyopathy being responsible for 45.31% of maternal deaths caused by cardiovascular diseases. The authors also reported that 37% of deaths were due to sudden cardiac death as a result of obesity. Of the 15.62% cases of SCD caused by hypertrophic cardiomyopathy, half were secondary to arterial hypertension, substance use, or valvular diseases. A worrying fact reported in the study is that of all sudden cardiac deaths, 10.93% were caused by pulmonary hypertension and 7.81% by aortic dissection.

Numerous studies have highlighted risk factors for SCD in pregnant women, including substance use (drugs, alcohol, tobacco), obesity, hypertension, preeclampsia, and diabetes. A common cause of pregnancy-related maternal deaths in low-income countries is rheumatic heart disease [2,72,73,74,75]. In their review and meta-analysis, Saccone G. et al. [76] identified an increased risk of maternal mortality in pregnant women over 40 years of age (advanced maternal age) compared to pregnant women younger than 40 years of age.

Table 1 summarizes the causes, etiology, and risk factors of sudden maternal death.

### 2.3. New Perspectives on Sudden Cardiac Death in Pregnancy

The United Nations Maternal Mortality Estimation Interagency Group (UN MMEIG) estimates a global maternal mortality rate of 223 deaths per 100,000 live births for the year 2020, with many of these deaths having preventable causes. For 2030, the target is to reduce the global maternal mortality rate to less than 70 per 100,000 live births. Another goal for 2030 is that no country should report more than 140 maternal deaths per 100,000 live births [77].

Recent data indicate that cardiovascular diseases, including cardiomyopathies, myocardial infarction, and stroke, account for more than 33% of pregnancy-related deaths in the United States [78]. An alarming fact is that a substantial proportion of these deaths are preventable. The Centers for Disease Control and Prevention (CDC) reported that over 80% of pregnancy-related deaths between 2017 and 2019 were preventable, and these deaths could be reduced or even eliminated by implementing rigorous guidelines in the care and follow-up of pregnant women [79].

To reduce the number of maternal deaths caused by cardiovascular diseases, the implementation of specialized multidisciplinary teams has been proposed. These teams typically comprise cardiologists, obstetricians, anesthesiologists, and nursing staff, all collaborating to manage high-risk pregnancies more effectively. Such coordinated care models have the potential to reduce maternal mortality and morbidity by ensuring comprehensive management of cardiovascular complications during pregnancy [80].

Another pillar through which maternal mortality can be reduced is the implementation of programs that lead to a growing emphasis on enhancing awareness and education among healthcare providers regarding the recognition and management of cardiovascular symptoms in pregnant patients. Early detection and timely intervention are crucial, as they can significantly improve outcomes for both mothers and infants.

### 2.4. Applications of Molecular Biology in Sudden Cardiac Death in Pregnancy

Molecular biology techniques are proving their effectiveness in an increasing number of medical fields and are gaining ground even in forensic medicine. Methods such as PCR (polymerase chain reaction), DNA sequencing, or NGS (next generation sequencing) can be utilized in “molecular autopsy”, which holds particular value in cases of sudden death where the forensic autopsy has not identified a cause of death (negative autopsies). In this context, susceptibility genes can be analyzed, such as KCNQ1, KCNH2, KCNE1, and KCNE2, which are involved in long QT syndrome, the RYR2 gene implicated in catecholaminergic polymorphic ventricular tachycardia type 1, or the SCN5A gene associated with Brugada syndrome [81,82].

In the case of cardiomyopathies, genes have been identified whose defects can lead to malignant rhythm disturbances before macroscopic changes in the heart become apparent [83]. Regarding hypertrophic cardiomyopathy, there are two key genes involved in encoding cardiac myosin-binding protein C and cardiac beta-myosin heavy chain. These genes (MYBPC3 and MYH7) are responsible for approximately 60% of hypertrophic cardiomyopathies [84]. A major role in the pathogenesis of arrhythmogenic cardiomyopathy is played by the genes PKP2, DSP, DSG2, and DSC2, which together are responsible for approximately 55–60% of all arrhythmogenic cardiomyopathies. In arrhythmogenic cardiomyopathies, there is a progressive replacement of myocardial tissue with fibro-fatty tissue. This primarily affects the right ventricle but can also involve the left ventricle, potentially leading to malignant arrhythmias and SCD [85].

Dilated cardiomyopathy is another possible etiology of SCD, consisting of dilatation of the left ventricle or both ventricles without the existence of coronary artery abnormalities, with consequences for cardiac contractility. It is well known that the vast majority of dilated cardiomyopathies are due to alcohol, drug use, myocarditis, or endocrine disorders. However, there is an important percentage of approximately 40% of dilated cardiomyopathies that are genetically determined. Mutations in genes encoding the cytoskeleton or sarcomeric proteins of cardiomyocytes are responsible for the occurrence of familial dilated cardiomyopathies [86]. The main genes involved in the pathogenesis of familial dilated cardiomyopathies are TTN, LMNA, and MYH7, which are responsible for approximately 35% of all familial CMD.

Genetic analysis of all the genes involved in the occurrence of cardiovascular diseases can significantly decrease the rate of negative autopsies if they are tested in cases of deaths in young people, especially when there is suspicion of an arrhythmia as the cause of death, including the case of maternal cardiac deaths. In addition, the identification of mutations in the genes involved in these diseases should lead to genetic testing of the first-degree relatives of the deceased person in order to establish rigorous measures to prevent SCD.

### 2.5. Prevention of SCD and Detection of Incipient Ischemia

Acute myocardial infarction can be diagnosed postmortem in conditions of survival of at least 6 h from the onset of ischemic injury by classical histopathological methods using the usual hematoxylin-eosin staining, but in conditions where death occurs within a few minutes or in the first hours, this represents a real challenge for forensic doctors. In recent years, the use of immunohistochemical markers has become imperative for the diagnosis of myocardial lesions.

In studies such as that of Campobasso C.P. et al. [87] and that of Sabatasso S. et al. [88], immunohistochemical markers are mentioned that have proven their usefulness in determining acute myocardial ischemia. Among them were listed C5b-9, FN (fibronectin), MB (myoglobin), connexin 43, cytochrome c, TUNEL, and cTnI. Among these, the markers that appear earliest in ischemia are connexin 43, JunB, and cytochrome c, which become positive within 30 min of ischemia onset. At approximately one hour of ischemia, fibronectin and myoglobin are positive, followed by troponins I and T, TUNEL, and C5b-9 (≤2 h) [89].

Although early detection of ischemia is essential in the prevention of SCD, a crucial step is the detection of coronary atherosclerosis in its early stages. In addition to imaging methods that have proven useful in the diagnosis of coronary atherosclerosis, serum levels of certain proteins or genes are gaining more and more ground.

MicroRNAs play a critical role in pathophysiological processes, such as cell adhesion, proliferation, lipid uptake, and inflammatory mediator production, offering valuable insights into coronary atherosclerosis and potential therapeutic approaches. They can be targeted as diagnostic, prognostic, and therapy response biomarkers for cardiovascular diseases, especially with the discovery that miRNAs can be detected in circulating blood. Cholesterol homeostasis, essential for cellular function, is regulated by lipoproteins like low-density lipoprotein (LDL) and high-density lipoprotein (HDL), with imbalances in these lipoproteins contributing to coronary atherosclerosis. Recent advances in understanding genes that regulate LDL and HDL levels have enhanced knowledge of plasma lipoprotein regulation. Notably, miR-122, a liver-enriched miRNA, regulates cholesterol and fatty acid production, playing a key role in lipoprotein homeostasis. Additionally, miR-223 and miR-27b influence cholesterol and lipoprotein metabolism through the post-transcriptional regulation of genes like hmgs1, sc4mol, and srb1, leading to altered HDL-C and cholesterol levels [90].

An important role in the pathogenesis of atherosclerosis is played by the genes ALB, SHBG, APOC, APOC3, APOC4, and SAA4, of which APOC3 and APOC4 make a major contribution to the occurrence of atherosclerosis and also to insulin resistance and type 2 diabetes [91,92,93].

### 2.6. Sudden Maternal Death—Autopsy Findings in Our Forensic Service

#### 2.6.1. Case Presentation No. 1

In this article, we present the case of a young mother aged 30 years in her 40th week of pregnancy, with no known pathological history, non-smoker and non-alcoholic, and VIIIG IVP. All four previous births were physiological, at term, with babies whose birth weights were between 4050 and 5300 g. The patient presented to the obstetrics and gynecology outpatient clinic at 40 weeks of gestation for a routine check-up, complaining of a cough. The clinical examination did not reveal any pathological changes. The patient’s BP was 120/70 mmHg, without painful uterine contractions. The patient was allowed to go home with a recommendation for a pulmonological consultation, but 8 h later, the pregnant woman was found in cardio-respiratory arrest (asystole) at home. Resuscitation maneuvers were instituted according to the protocol, but without response. Laboratory tests taken during resuscitation revealed red blood cells = 3.5 million, hematocrit = 28.9%, platelets = 64,000 cell/µL, hemoglobin = 9.1 mg/dL, leukocytes = 8500/µL, lymphocytes = 58.9%, granulocytes = 34.8%, hs-cTnI = 37.6 ng/L, and D-Dimers = >5 µg/mL.

In Romania, the legislation given by the Criminal Procedure Code stipulates that it is mandatory to order a forensic autopsy in all cases where the cause of death is unknown; thus, in the case of maternal deaths that occur at home, a forensic autopsy is mandatory.

The necropsy revealed the following: Caucasian female, normosthenic constitution, height 167 cm, weight 68 kg. External examination revealed cyanosis of the cephalic extremity, uterine fundus height 3 cm below the xiphoid process, and no signs of deep venous thrombosis (the circumference of the calves and thighs being equal bilaterally, measured at the same level). No traumatic lesions were found. Internal examination identified bilateral pleurisy (250 mL serous citrine fluid in the right pleural cavity and 150 mL in the left pleural cavity). Lung sectioning revealed signs of acute pulmonary edema, zonal bronchiolitis, and apically right posteriorly, two areas of condensation of the pulmonary parenchyma were found, with a nodular appearance that protruded subpleurally by 2/1.5/1.5 cm, on section with a carnified appearance.

The pericardial sac contained 50 mL of serous citrine fluid. The heart was globally enlarged in size, weighing 400 g, with both the right and left heart cavities significantly dilated. The valve apparatus was supple, smooth, and normally shaped, the epicardium was smooth and glossy, and the parietal endocardium was diffusely thickened and opaque, with increased consistency. The maximum thickness of the left ventricular wall was 22 mm, the interventricular septum was 20 mm, and the right ventricle was 3 mm. Within the thickness of the ventricular myocardium, numerous fibrous, diffuse, whitish bands were observed. The coronary arteries were patent, with occasional fibro-hyaline plaques under the intima.

The ovoid, pregnant uterus measured 35/33 cm. A transverse section of the suprasymphyseal antero-inferior uterine prerectus revealed a normal amount of amniotic fluid with a serous-hemorrhagic appearance, a dead male fetus in utero, in left occipital cranial presentation, with a loose cord loop around the neck. The placenta was located on the left postero-lateral wall of the uterus, without visible macroscopic lesions, with maturation grade III. The fetus weighed 3900 g and was 51 cm long, with somatic development corresponding to a full-term fetus, without congenital malformations.

**Figure 1:**
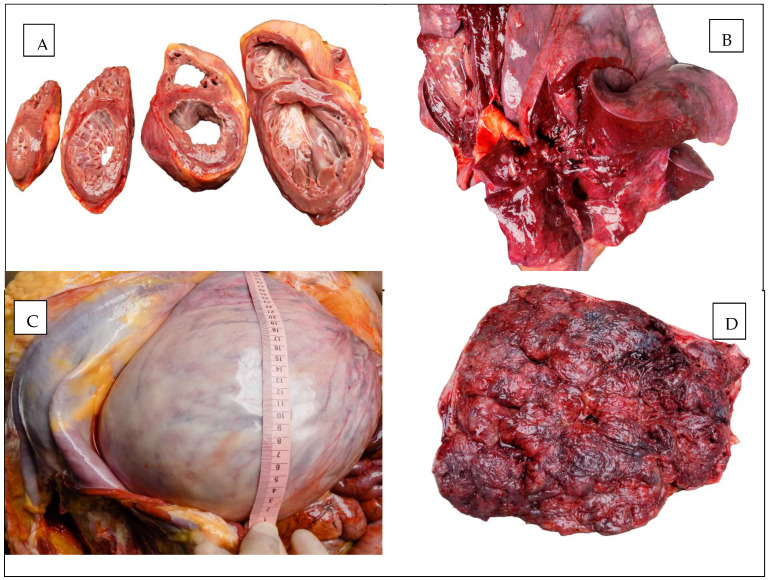
Autopsy aspect: (**A**) The heart. (**B**) The lung. (**C**) The uterus. (**D**) The placenta.

The macroscopic appearances observed in the heart, lungs, and uterus are illustrated in Figure 1.

For the histopathological examination, three fragments of lung parenchyma and three fragments of heart were collected, including a fragment from the left ventricle, a fragment from the interventricular septum, and a fragment from the right ventricle. At the level of the lung parenchyma, the following were highlighted: acute pulmonary edema, focal pulmonary emphysema, changes suggestive of interstitial pneumonia with areas of conjunctival organization of the lung parenchyma, and acute focal bronchiolitis.

**Figure 2:**
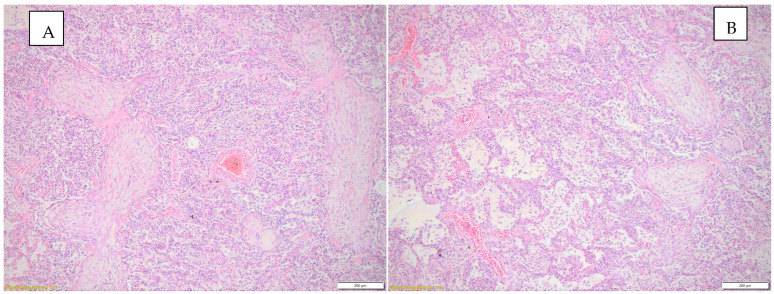
(**A**) Histopathological changes observed in the lung parenchyma (inflammatory infiltrate, interstitial pneumonia—conjunctival organization), HE, objective 10X. (**B**) Histopathological changes observed in the lung parenchyma (emphysema, inflammatory infiltrate, interstitial pneumonia), HE, objective 10X.

Figure 2 highlights the histopathological changes observed in the lung parenchyma.

**Figure 3:**
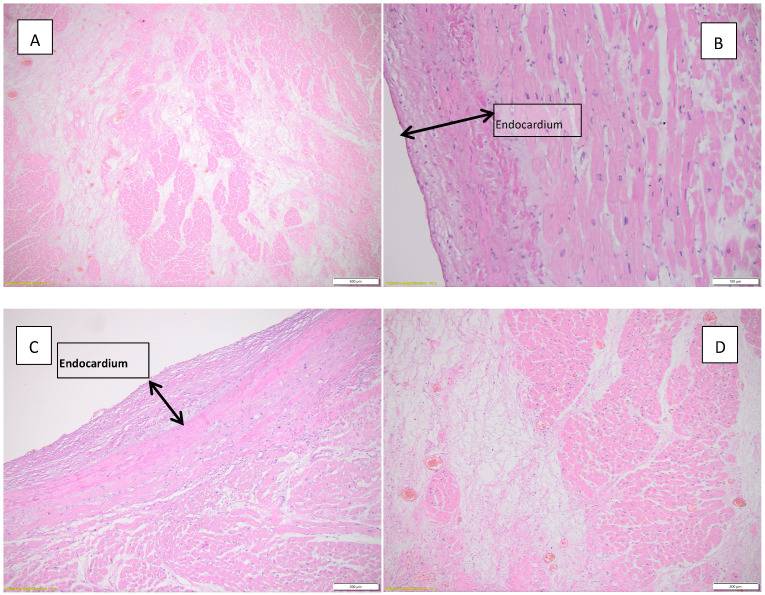
Histopathological findings observed in the heart. (**A**) Acute myocardial infarction in the process of organization, HE, objective 4X; (**B**) endocardial fibroelastosis, HE, objective 20X; (**C**) endocardial fibroelastosis, HE, objective 10X; (**D**) myocardial infarction in the process of conjunctival organization can be seen in the left half of the image; HE, objective 10X.

Figure 3 highlights histopathological changes observed in the heart.

The toxicological examination performed on the blood collected during the autopsy was negative.

Following the necropsy, toxicological tests, and microscopic examination, it was concluded that death was non-violent (pathological) and due to acute myocardial infarction occurring in an organism with chronic cardiac pathology, i.e., hypertrophic cardiomyopathy, myocardial lipomatosis, and endocardial fibroelastosis.

#### 2.6.2. Case Presentation No. 2

The second case corresponds to a 42-year-old pregnant woman, with a bivitelline, monochorial biamniotic twin pregnancy in the 37th week of gestation, with no known pathological history, 3G2P, who was found at home in cardio-respiratory arrest after a convulsive crisis. The arriving crew initiated resuscitation maneuvers, and the patient responded to them. She was orotracheally intubated and mechanically ventilated. On admission, she presented with GCS 3 points, respiratory rate 14/min, pulse 80 b/min, BP 110/50 mmHg, and Sat O_2_ = 82%. On admission, laboratory tests were performed with the following values: WBC (white blood cells) = 29.1 × 10^3^/μL (3.5–10 × 10^3^/μL), LYM (lymphoytes) 7.0 × 10^3^/μL (0.9–5 × 10^3^/μL), GRA (granulocytes) 20.8 × 10^3^/μL (1.2–2.8 × 10^3^/μL), lactate 12.7 mmol/L (0.6–2.2 mmol/L), hs-cTnI (high-sensitivity troponin I) > 1350 ng/L, CKMB (creatine kinase MB) 170 U/L (0–25 U/L), GOT (aspartate aminotransferase) 64 U/L (8–38 U/L), and AMYL (amylase) 250 U/L (37–125 U/L). After admission, an emergency cesarean section under general anesthesia and IOT was carried out, and two dead female fetuses were extracted, weighing 2140 g, L = 45 cm, and 2600 g, L = 47 cm.

After 4 h of hospitalization, a second episode of cardiorespiratory arrest occurred. Cardiopulmonary resuscitation maneuvers were initiated, but without success.

The autopsy revealed a Caucasian woman, overweight, 160 cm tall, weighing 88 kg, with a BMI of 34.37. The external examination revealed cyanosis of the cephalic extremity, and the height of the uterine fundus was 15 cm lower than the xiphoid process. No signs of deep venous thrombosis were evident (the circumference of the calves and thighs being equal bilaterally, measured at the same level). Traces of multiple venous punctures were identified, with a horizontal post-cesarean surgical wound measuring 17 cm long, supple, faced with six sutures, 1 cm lower than which, in the middle third, a drain tube was sutured to the skin, through which reddish bloody fluid was externalized. The internal examination, at the level of the thoracic cavity, revealed a minimal amount of serous citrine fluid in both pleural cavities. When the lungs were sectioned, parenchyma of increased consistency was found, with a marbled appearance.

The pericardial sac contained 7 mL of serous citrine fluid. The heart measured 12.5/12.5/5.3 cm, weighed 460 g, and the cavities were dilated bilaterally. The valvular apparatus was supple, smooth, and normally conformed; the bicuspid and tricuspid valves were free, triangular, soft, thin, and mobile; the chordae tendineae were thin; and the papillary muscles were conical in shape, slightly thickened on the left. The epicardium was smooth and transparent, and at the subendocardial level of the antero-septal wall corresponding to the base of the papillary muscles, a reddish blood infiltrate was found in an area of 2/2/0.1 cm with petechial appearance. The myocardium was brown–chestnut in color, pale, and with slightly reduced consistency, with the maximum thicknesses of the left ventricle being 20 mm, the interventricular septum 20 mm, and the right ventricle 3 mm. Coronary arteries with bilaterally permeable coronary ostia, subintimal with rare, hard, isolated fatty deposits, protruded into the lumen at the level of the incipient portion of the right and left coronary branches over a length of about 10 mm. The lumen of the left anterior descending artery was slightly diminished, with a short path of approximately 40 mm.

The ovoid uterus had dimensions of 150/180/50 mm with shiny, transparent serosa at the level of the lower uterine segment. On the anterior wall, there was a horizontal Monroe–Kerr incision, 90 mm long, with 10 suture threads/10 stitches. When the uterus was opened, the cavity was empty, without pathological content, and the uterine wall was pinkish in color, with a thickness of 2.5–3 cm.

**Figure 4:**
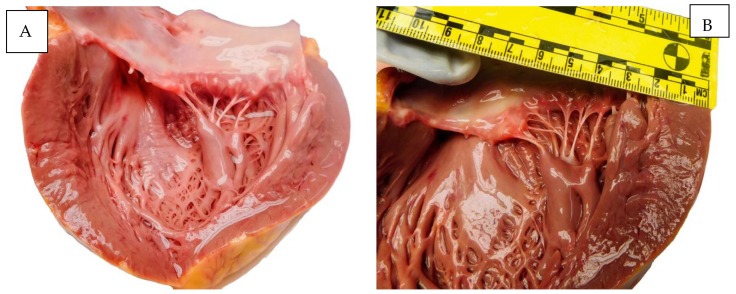

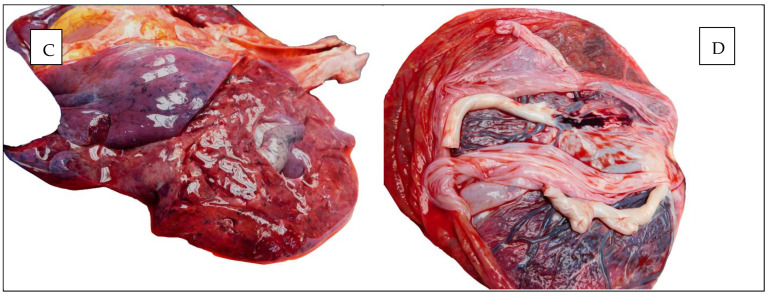
Autopsy aspects. (**A**,**B**) The heart. (**C**) The lung. (**D**) The placenta.

Figure 4 illustrates macroscopic appearances found at autopsy.

Five fragments of lung tissue and two fragments of the heart were collected for microscopic examination.

Histopathological examination of the lung tissue (five fragments) revealed mononuclear inflammatory infiltrate (lymphocytes, plasma cells) as well as neutrophils in abundant quantity. Some alveolar areas showed eosinophilic, fine-granular intraluminal material, and, in some places, groups of macrophages were present. Some capillaries contained megakaryocytes, and other vascular structures had optically empty vacuoles. In some areas, the alveolar spaces were collapsed, having an atelectasis appearance.

**Figure 5:**
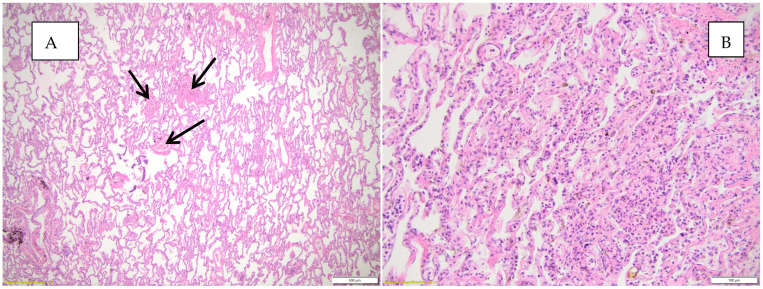
Histopathological appearances observed in the lung parenchyma. (**A**) Abundant mononuclear inflammatory infiltrate and microthrombi (black arrows), atelectasis HE, objective 20X; (**B**) abundant mononuclear inflammatory infiltrate, collapsed, atelectasis alveoli, HE, objective 20X.

Figure 5 highlights the histopathological changes observed in the lungs.

The histopathological examination of the heart revealed borderline myocarditis (according to Dallas criteria), focal areas suggestive of acute myocardial ischemia, and myocardial fiber hypertrophy. The myocardial fibers had increased size, and the nuclei were enlarged in volume. Interstitially, moderate, diffusely dispersed neutrophilic and lympho-plasmacytic inflammatory infiltrate was observed. Areas with interstitial edema were identified, with myocardial fibers without nuclei, some fragmented, being isolated with a wavy appearance. The vascular structures had leukocytosis.

The diagnosis of borderline myocarditis was made according to the Dallas criteria updated by the European Society of Cardiology in 2013. For the diagnosis of active myocarditis, the following histopathological changes are required: the presence of mononuclear inflammatory infiltrate, signs of necrosis, and non-ischemic degeneration of cardiomyocytes. For the diagnosis of granulomatous myocarditis, the following histopathological changes are required: scattered inflammatory infiltrate, without cardiomyocyte involvement. The inflammatory infiltrate must contain more than 14 leukocytes/mm^2^, of which at least 4/mm^2^ must be mononuclear [94,95].

**Figure 6:**
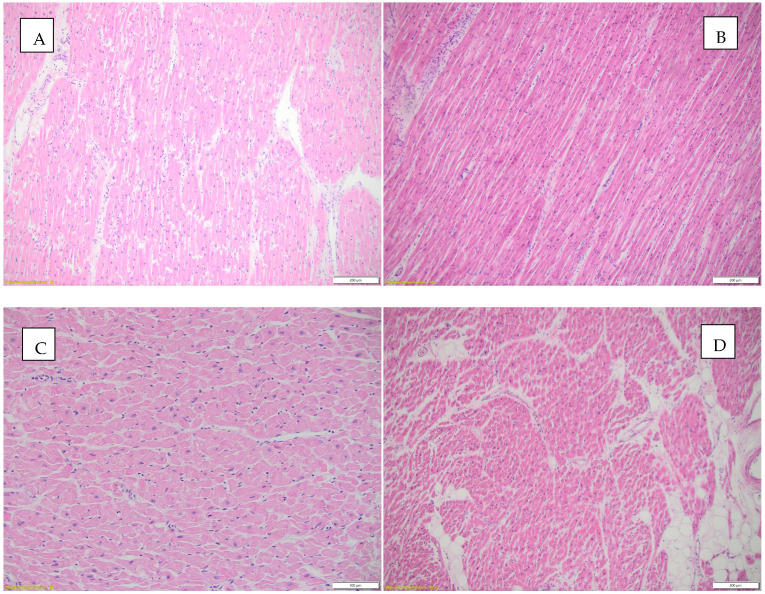
Histopathological aspects observed in the heart. (**A**) Borderline myocarditis (according to Dallas criteria) and hypertrophied myocardial fibers, HE, objective 10X; (**B**) inflammatory infiltrate and interstitial edema, HE, objective 10X; (**C**) focally fragmented cardiac fibers are observed, some without nuclei, others with a wavy appearance, HE, objective 20X; (**D**) interstitial edema, inflammatory infiltrate, and focally present intracardiac adipose tissue, HE, objective 4X.

Figure 6 illustrates the histopathological appearances observed in the heart.

A toxicology test was performed and was negative.

A lung tissue fragment was sent to a reference center (Cantacuzino National Institute for Medical–Military Research and Development, Bucharest) for bacterial cultures, microscopy, biochemical tests, and molecular biology tests. Following bacterial cultures, microscopic examination, and biochemical tests, the Neisseria meningitidis serogroup B strain was isolated. SARS-CoV-2 viral RNA testing was performed by real-time PCR, the result being negative. Genomic determinations were also performed for respiratory viruses from necropsy lung fragments using real-time RT-PCR multiplex for influenza virus types A and B, respiratory syncytial virus, parainfluenza viruses, coronaviruses Oc43, 229 E, and NL 73, metapneumovirus, rhinovirus, bocavirus, enterovirus, and adenovirus, all of which were negative.

The forensic autopsy concluded that the death was non-violent (pathological). It was due to multiple organ-system failure as a consequence of disseminated intravascular coagulation in a pregnant woman with resuscitated cardio-respiratory arrest. All of these occurred in the course of a fulminant meningococcal infection (Neisseria meningitides serogroup type B), with pulmonary (severe interstitial pneumonia) and myocardial (borderline interstitial myocarditis) involvement in a patient with an ongoing twin pregnancy with a gestational age of 37 weeks.

## 3. Discussion

Endocardial fibroelastosis (EFE) is a rare heart disease characterized by heart failure due to thickening of the ventricular endocardium resulting from the proliferation of collagen and elastic fibers [96,97]. It generally occurs in infants and children presenting primary etiology (absence of causal factors) or secondary to congenital heart malformations, aortic stenosis, or coarctation, with ventricular septal defects frequently leading to death in the first years of life [98,99,100]. According to literature data, EFE showed a dramatic decrease in the number of cases in developed countries after 1980, although a significant peak between 2004 and 2005 was reported in England, Galicia, USA, following a mumps epidemic that affected young people, this representing a significant risk factor for the development of fibroelastosis [101]. Another etiology of EFE is attributed to “idiopathic heart disease”. EFE in adults is reported extremely rarely, being identified postmortem following autopsy and histopathological examination [102,103]. The study by Pastor Quirante F.A. et al. [104] describes a case of sudden cardiac death in a 43-year-old female adult with no pathological history, in whom the necropsy histopathological examination revealed the presence of fibroelastosis, but associated with hypothyroidism. To our knowledge, this is the first case of sudden cardiac death in pregnant women due to fibroelastosis reported in the literature.

The second presented case is also a rare one. It is known that myocarditis is an important cause of SCD, but there are very few cases reported in the literature in which borderline myocarditis is considered the cause of sudden death. In general, these were determined by viruses such as parvovirus B19 [105]. Following extensive searches in the literature, we found no cases in which infection with Neisseria meningitidis led to maternal deaths through multiple organ failure on the back of a fulminant infection. Neisseria meningitidis is a diplococcus, gram-negative, encapsulated, aerobic bacterium associated with increased mortality and morbidity worldwide, affecting children and young people and causing meningococcal meningitis [106,107,108]. In adults, it colonizes 5–15% of the normal flora of the nasopharynx, the annual incidence of meningococcemia in Europe being one case per 100,000 of the six serogroups, the most frequent being serogroup B [109,110,111,112]. Atypical cases caused by Neisseria meningitis causing pneumonia, cardiac involvement (pericarditis, myocarditis), and laryngitis are, according to literature data, extremely rare and mainly in elderly patients with associated comorbidities [113].

## 4. Conclusions and Future Directions

Sudden maternal death is a health problem in both developed and developing countries. SCD often has preventable causes; thus, reducing maternal mortality should be a priority in the field of public health. Early identification of risk factors involved in the occurrence of cardiovascular diseases and their treatment (atherosclerosis, hypertension, arrhythmias, etc.), both prenatally and during pregnancy, as well as postpartum, is essential. At the same time, determining the cause of sudden death in pregnant women through genetic determinations and molecular biology techniques (where there is suspicion of diseases with genetic determinism) is absolutely necessary to prevent the occurrence of sudden deaths among close relatives.

We consider it appropriate to document cardiac changes that may occur during pregnancy by performing cardiovascular screening during pregnancy and, subsequently, during gestation, with careful monitoring in order to identify the causes of sudden maternal deaths. Also, the establishment during pregnancy of multidisciplinary teams comprising specialists in maternal–fetal medicine, cardiology, cardiovascular surgery, and anesthesia represents an extremely important prevention method in reducing SCD in pregnant women.

## Figures and Tables

**Table 1:** Causes, etiology, and risk factors of sudden maternal death.

Causes and Etiology		Risk Factors
Hypertrophic cardiomyopathy	Amniotic fluid embolism	Obesity
Restrictive cardiomyopathy	Atherosclerosis	Advanced maternal age
Dilated cardiomyopathy	Valvular diseases	Multiparity
Arrhythmogenic right ventricular cardiomyopathy	Congenital heart diseases	Race
Aortic and coronary artery dissection	Endocarditis	Chronic arterial hypertension
Acute myocardial infarction	Intracoronary thrombus without atherosclerosis	Thrombophilia
Myocarditis	Coronary artery spasm	Preeclampsia
Myocardial fibrosis		Diabetes

## Data Availability

The original contributions presented in this study are included in the article; further inquiries can be directed to the corresponding authors. The data presented in this study are available on request from the corresponding author due to privacy, legal reasons, and ethical reasons.
